# Effective practices and transdisciplinary team-based approaches in home palliative care for terminal cancer patients: a qualitative descriptive study

**DOI:** 10.1186/s12904-026-02102-3

**Published:** 2026-04-17

**Authors:** Miyuki Yoshida, Eiko Yamauchi, Keiko Suyama

**Affiliations:** Department of Nursing, Graduate School of Medicine, Ehime University, Toon, Japan

**Keywords:** Terminal cancer, Home-based palliative care, Transdisciplinary team care, Team practice, Qualitative research

## Abstract

**Background:**

In Japan, approximately 60% of patients with cancer prefer to spend their final days at home, yet only 11.8% die at home. Contributing factors include uneven distribution of community resources and difficulties in multidisciplinary collaboration. We aimed to qualitatively identify key practices in home-based palliative care that should be shared among professionals within a transdisciplinary team approach, which may offer solutions to these challenges.

**Methods:**

Between November 2021 and March 2022, semi-structured interviews were conducted with 13 professionals—physicians, nurses, care managers, care workers, pharmacists, and medical social workers—affiliated with institutions that provide more than 12 cases of home-based end-of-life care annually. We used reflexive thematic analysis to code the data, extracted subcategories and main categories, and inductively developed the themes.

**Results:**

We identified 625 codes, 66 subcategories, and nine categories, which were integrated into three themes. Theme 1 (Iterative Communication & Coordination) described repeated alignment of prognosis and care goals among teams, patients, and families, supported by real-time information sharing. It also illustrated how teams recalibrated care direction in response to subtle changes in patient conditions or family perspectives. Theme 2 (Family Readiness & Relational–Emotional Support) captured practices that strengthened caregivers’ psychological readiness and emotional stability by alleviating burden, responding sensitively to distress, and fostering meaningful connections. This theme also highlighted that caregiver needs fluctuate throughout the illness trajectory, requiring tailored and ongoing engagement from multiple professionals. Theme 3 (Proactive Preparedness & Resource Orchestration) encompassed anticipatory symptom management, coordination of financial/practical resources, and flexible cross-disciplinary role-sharing to ensure timely responses at home. Proactive preparation—such as securing medications, anticipating care gaps, and coordinating resources across institutions—played a crucial role in preventing crises and sustaining home care. Collectively, these integrated practices enabled resource-conscious, transdisciplinary support that contributed to the realisation of home deaths in resource-limited settings.

**Conclusions:**

These findings inform education and system design by clarifying how transdisciplinary teams integrate iterative communication, caregiver support, and proactive preparation to provide flexible, comprehensive care, even in resource-limited settings, thereby offering a foundation for developing transferable training frameworks and implementation strategies across diverse care contexts.

**Supplementary Information:**

The online version contains supplementary material available at 10.1186/s12904-026-02102-3.

## Introduction

Cancer remains the leading cause of death in Japan [1], and approximately 60% of patients with cancer express a desire to spend their final days at home [2]. The preference for home-based care among terminal patients with cancer often stems from a desire to maintain dignity, live as ordinary individuals within a familiar environment, and accept themselves as recipients of care [3, 4]. This wish is deeply personal and essential for many patients; however, only 11.8% of deaths occur at home [5], indicating a substantial gap between patients’ preferences and actual outcomes.

To realise home-based end-of-life care, it is necessary to identify the contributing factors and establish supportive systems. Medical factors include the availability of emergency response systems [6, 7], access to facilities for emergency hospitalisation [7, 8], and the development of trusting relationships with healthcare providers [4, 8]. Patient- and family-related factors include a stated wish for home death [9–13] and the presence of sufficient family caregiving capacity [8, 9, 13].

These factors influence both the quality and sustainability of home-based end-of-life care. Well-established emergency response systems and accessible hospitalisation pathways reduce anxiety during acute changes, thereby stabilising the delivery of home care. Trusting relationships with healthcare providers enable effective communication and shared decision-making, ensuring care that aligns with patient values. In addition, a clearly expressed wish for home death provides direction for care planning, while adequate family caregiving capacity supports the continuous provision of daily care. Together, these conditions determine the sustainability of high-quality home-based palliative care until the end of life.

Previous studies have suggested that multidisciplinary and team-based approaches improve outcomes of home palliative care through several mechanisms, including shared decision-making, timely symptom management, and coordinated role allocation among professionals. These mechanisms have been associated with reduced emergency hospital admissions, improved symptom control, and increased concordance between patients’ preferences and place of death [14, 15].

Taken together, evidence suggests that the realisation of home-based end-of-life care for patients with terminal cancer requires a high-quality palliative care system delivered by a cohesive team, along with support that enhances family caregiving capacity and responsiveness to medical needs. However, such ideal teams are not consistently available across regions.

The population in Japan is rapidly declining, particularly in rural areas, with large regional disparities in the use of medical and long-term care services. Ito et al. [16] reported a five-fold regional disparity in access to home healthcare that was linked to the concentration of healthcare institutions and home care support clinics within industrial clusters. Moreover, the regional difference in the supply capacity of home nursing services is as high as eight-fold in mountainous areas and remote islands nationwide [17]. In particular, service availability is substantially lower in rural and mountainous areas than in urban areas. These regional disparities arise from the maldistribution of healthcare resources, shortages of specialist providers, geographical barriers, and variability in the organisation of home care support systems. In rural and remote regions, the limited availability of home-visiting physicians and nurses reduces timely access to symptom management and emergency responses, directly affecting the feasibility of home-based end-of-life care. Comparable disparities have been reported internationally. For example, studies from Canada and the United States have shown that rural residents have reduced access to palliative care specialists, with higher rates of unplanned hospitalisations and a lower likelihood of home death [18, 19]. Collectively, these findings indicate that equitable access to home healthcare is a common international challenge rather than a Japan-specific issue.

Accordingly, knowledge of regional disparities in Japan and the development of strategies to mitigate them have international relevance. Where specialist availability is limited, flexible role-sharing within teams has been proposed as an effective strategy to compensate for resource constraints [20, 21]. Therefore, in resource-limited areas, a transdisciplinary approach is particularly crucial to meet the support needs required to enable home death. Implementing such an approach entails sharing common goals, developing a clear understanding of the collective practices and roles required to achieve them, and coordinating role allocation within the team.

Outcome measures for palliative care have been developed in previous studies. Claessen et al. [22] proposed 33 indicators for patient care and 10 indicators for caregiver support in the Netherlands, while Hearn and Higginson [23] developed 12 core outcome measures in the UK and Scotland. These metrics cover key areas such as symptom management, quality of life, communication, decision support, and caregiver wellbeing, directly reflecting the core elements of high-quality home palliative care. These outcome measures are not only indicators of quality of care, but also important prerequisites for facilitating death at home. Therefore, they represent a common quality goal for multidisciplinary teams. In response, Morita et al. [24] presented process indicators for evaluating palliative care practices; however, their target population was limited to doctors and nurses in hospitals and did not include welfare professionals [such as care managers (CMs) and certified caregivers] who assist patients in their daily lives. In addition, regional linkage indicators have been developed [15], although they measure system-level alignment rather than the specific practices of individual multidisciplinary teams. To date, relatively few studies have comprehensively examined home palliative care practices delivered by an integrated, multidisciplinary team.

Therefore, this study aimed to elucidate the concrete practices, collaborative processes, and role-sharing mechanisms through which transdisciplinary teams enable home-based end-of-life care for patients with terminal cancer, using a qualitative descriptive design. By articulating these empirically derived practices, the study seeks to offer a practice-oriented and analytically transferable framework to inform the implementation of transdisciplinary teams across diverse regional and organisational contexts.

## Methods

### Study design

A qualitative descriptive study design was employed using individual interviews. This approach was selected to generate new knowledge and insights into the practices of interprofessional, integrated teams in home-based palliative care using reflexive thematic analysis [25, 26]. This study followed the Consolidated Criteria for Reporting Qualitative Studies (COREQ) checklist [27] [see Additional file 1].

### Participant selection criteria

We employed purposive sampling to recruit multidisciplinary professionals with experience in supporting home deaths among patients with terminal cancer. Participants were healthcare professionals who had either published practice reports on home-based palliative care for patients with terminal cancer in academic or professional journals or were affiliated with institutions that manage at least 12 cases of home-based end-of-life care annually. Eligible participants were professionals engaged in home palliative care as part of a multidisciplinary team supporting home death for patients with terminal cancer and who provided informed consent to participate in the study. Only individuals able to communicate in Japanese were included. The exclusion criteria were as follows: individuals with no experience in providing home-based end-of-life care for patients with terminal cancer; those who worked independently without belonging to a multidisciplinary team; those who did not provide informed consent; those whose roles were unrelated to home-based palliative care (e.g., healthcare administrators); and those with insufficient practical experience to contribute relevant information to the study.

In terms of professional backgrounds, the study participants included physicians, nurses, CMs, certified care workers, pharmacists, and medical social workers (MSWs) holding a social work qualification. Institutions with a record of 12 or more home-based end-of-life care cases per year were identified using publicly available information from regional health bureaus and prefectural government websites.

The threshold of 12 cases was determined based on the criteria for Enhanced Home-Visit Nursing Stations Type I and II in Japan [28]. Type I requires either 20 or more annual home deaths (regardless of diagnosis) or 15 or more deaths plus continuous care for four children with severe illness. Type II requires either 15 or more annual deaths or 10 or more deaths plus care for three children with severe illness. Based on these standards, non-cancer cases were excluded, and institutions managing at least 12 cancer-related home deaths per year (averaging one per month) were considered to possess sufficient experience in end-of-life care for patients with cancer.

In Japan’s home care system, CMs and MSWs play essential roles. CMs develop care plans under the long-term care insurance system for older adults, while MSWs provide support for social issues. Both professionals are key coordinators for daily living support and medical collaboration for patients and families and are central to the delivery of multidisciplinary home-based palliative care.

None of the eligible participants refused to participate in the study. All interviews were conducted in Japanese.

The sample size was determined in line with the principles of reflexive thematic analysis, with adequacy assessed based on the richness and depth of the data rather than the achievement of data saturation. The aim was not to exhaust all possible codes within each profession but to generate sufficiently information-rich accounts to support meaningful pattern development across the dataset. As this study sought to understand integrated multidisciplinary practice as a whole, profession-specific saturation was not required.

### Data collection

Data were collected between November 2021 and March 2022 through individual interviews with expert professionals from various disciplines. Although focus group interviews were initially considered as a method for exploring team practices, individual interviews were ultimately chosen. This decision was based on the following considerations specific to home-based palliative care: the team composition varies across patients; power dynamics between medical and welfare professionals in group settings may suppress the voices of welfare professionals; and detailed exploration of practices—particularly from underrepresented professions in the existing literature—was required.

The interviews were conducted during the COVID-19 pandemic, and participants were given the option to participate via a web conferencing platform. After obtaining informed consent, basic demographic and professional data were collected from each participant. This included age, gender, professional qualifications, years of experience in their primary profession, and the number of patients with terminal cancer they had supported through home-based palliative care and end-of-life care as members of multidisciplinary teams.

Semi-structured interviews were conducted using an interview guide [see Additional file 2]. Participants were asked to describe high-quality home-based palliative care practices provided by multidisciplinary teams that had successfully fulfilled the wishes of patients with terminal cancer to die at home. Specifically, they were asked to recall memorable cases in which patients, families, and team members were satisfied with the end-of-life care provided. Participants described the process from the patient’s expressed desire for home care through the provision of care at home to bereavement support. They also discussed their own practices, those of other team members, and the responses of patients and families. Furthermore, they were asked to reflect on how collaboration was achieved within the team, how challenges such as manpower shortages were managed, and which practices or coordination efforts were considered essential for achieving a satisfactory home death for all involved.

The interview guide was developed using broad, open-ended questions informed by the study objectives and prior research [6–24] on home-based end-of-life care. The guide was not anchored to a fixed theoretical framework; rather, it was designed to elicit participants’ experiential accounts across key domains, including patient care, family support, and multidisciplinary collaboration. This approach is consistent with reflexive thematic analysis, which values flexibility and openness to participants’ perspectives.

There was no prior relationship between the interviewers and participants. Before the interviews, participants were informed that the researchers were conducting the study independently and had no supervisory or clinical role in participants’ clinical practice or in the care they provided to patients.

Field notes were not used during or after the interviews.

The interviewer (M.Y.) is a Certified Nurse Specialist in Community Health Nursing and a registered nurse with over 20 years of clinical experience in home-based palliative care. She holds a PhD in Nursing and received formal training in qualitative research methods, including semi-structured interviewing and thematic analysis, during her doctoral program and through specialised workshops.

### Analysis

A qualitative descriptive study design based on Braun and Clarke’s six-phase framework of reflexive thematic analysis was employed [25, 26].

Phase 1 (data familiarisation) involved repeated reading of the verbatim interview transcripts by the interviewer to gain an overall understanding of the data while noting initial impressions and salient features.

Phase 2 (generating initial codes) entailed the identification of data segments relevant to the research objectives and the generation of initial codes. In accordance with a reflexive approach, coding was conducted with explicit awareness of the researcher’s interpretive position; participants’ accounts were concisely rephrased, and, where necessary, omitted subjects were supplemented without altering the original meaning.

Within this reflexive thematic analysis, themes were not considered entities that naturally emerged from the data; rather, they were understood as interpretative constructions developed through the researcher’s active engagement with the dataset.

Phase 3 (searching for candidate themes) involved the organisation of the initial codes into subcategories based on semantic similarity.

Phase 4 (reviewing themes) was conducted by two authors (M.Y. and E.Y.), who examined the coherence of subcategories and categories in relation to the entire dataset and assessed their conceptual adequacy.

Phase 5 (defining and naming themes) involved iterative and interpretive discussions within the research team, through which categories were further abstracted into higher-order themes, and analytical boundaries between themes were refined and clarified.

Phase 6 (producing the report) involved the presentation of the findings using a hierarchical analytical structure (codes → subcategories → categories → themes), which reflected the interpretive nature of reflexive thematic analysis and provided a coherent conceptual framework for reporting the results.

Researcher reflexivity was explicitly considered throughout the analytical process. The primary researcher (M.Y.) recognised that her professional background and values, particularly her emphasis on autonomy, family involvement, and interdisciplinary collaboration, could influence coding decisions and theme development.

As an example of reflexive analysis, the primary researcher reflected on the possibility that her long-standing involvement in home-based palliative care might predispose her to attend more readily to caregiver burden and support practices. This issue was examined through discussions within the research team, during which themes were revisited to ensure that nursing perspectives were not overemphasised and that interpretations derived from other professional viewpoints were adequately represented.

The primary researcher also reflected on how her familiarity with the cultural norms and collaborative practices characteristic of Japanese home-based palliative care could lead to certain practices being taken for granted. To critically examine these influences, reflexive notes were maintained throughout the analysis, and iterative discussions within the research team were used to continually situate and contextualise the researcher’s interpretive position.

Discussions among co-researchers were not intended to achieve consensus on a single “correct” interpretation; rather, they were conducted to explore how each researcher’s professional background and clinical experience informed the analytical process. To enhance the overall rigour of the study, two authors independently coded the dataset, and discrepancies were resolved through discussion; this approach prioritised analytical depth over conventional positivist notions of intercoder reliability.

The findings were translated into English by a professional translator. No qualitative data analysis software was used; all coding and categorisation were conducted manually.

### Ethical considerations

This study was approved by the Research Ethics Committee of the Graduate School of Health Sciences, Ehime University (Approval No.: Nursing 2021-17). Before participation, all participants received a written explanation outlining the purpose and objectives of the study, procedures for protecting personal information and privacy, limitations on data use, publication of research findings, and measures to prevent any disadvantages resulting from participation. Written informed consent was obtained from all participants.

To safeguard confidentiality, participants were instructed to avoid using identifiable names during interviews. Transcripts were anonymised by removing personal identifiers. Interviews were conducted in private environments where voices could not be overheard. Participants were informed that they were free to decline to discuss any topic they found uncomfortable, and every effort was made to create a relaxed and open atmosphere that encouraged voluntary and spontaneous responses. Interviewers maintained a passive yet attentive stance to support this approach.

Participants were assured of their right to withdraw from the study at any time. Transcription of the interview recordings was outsourced to a professional service provider under a confidentiality agreement, in accordance with Ehime University’s research regulations.

## Results

### Characteristics of the participants

The study included 13 professionals, including four men and nine women: three physicians, three home care nurses, two CMs, three certified care workers, one pharmacist, and one licensed MSW. The sample was selected using purposive sampling to recruit multidisciplinary professionals with substantial experience in supporting home deaths. Following the completion of all interviews, thematic analysis was conducted. The dataset of 13 interviews demonstrated adequate variation across professional roles and provided sufficient analytical depth to support robust theme construction within a reflexive analytical framework. The average interview duration of each interview was 72.8 min. Participants had a mean age of 55.8 years and an average of 25.5 years of experience in their primary profession. In the previous year, participants had managed an average of 33.8 home deaths (Table 1).

**Table 1:** Overview of Interview Participants

Participant	Profession	Sex	Age (years)	Experience (years)	Number of Home Deaths in Past Year	Interview Duration (minutes)
A	Physician	Man	70s	45	20	108
B	Physician	Man	60s	40	200	43
C	Physician	Man	70s	34	40	72
D	Visiting Nurse	Woman	40s	25	16	85
E	Visiting Nurse	Woman	50s	31	30	69
F	Visiting Nurse	Woman	60s	45	23	102
G	Care Manager	Man	50s	9	46	38
H	Care Manager	Woman	40s	21	14	108
I	Care Worker	Woman	30s	5	4	52
J	Care Worker	Woman	60s	5	11	48
K	Care Worker	Woman	50s	16	8	60
L	Pharmacist	Woman	50s	30	15	87
M	Licensed MSW	Woman	40s	26	12	74
Mean ± SD			55.8 ± 11.6	25.5 ± 13.9	33.8 ± 51.4	72.8 ± 24.2

Values are shown as mean ± standard deviation (SD)

### Qualitative findings

Through the analysis, we identified 625 codes, 66 subcategories, and nine main categories. Through iterative analytical abstraction, these nine main categories were integrated into three higher-order themes representing the core dimensions of transdisciplinary home palliative care practice: (1) Iterative Communication & Coordination, (2) Family Readiness & Relational–Emotional Support, and (3) Proactive Preparedness & Resource Orchestration (Table 2). Each theme comprises functionally related categories that collectively illustrate how integrated multidisciplinary teams sustain home-based end-of-life care.

**Table 2:** Multidisciplinary Practices for High-Quality Home Palliative Care Enabling Home Death in Cancer Patients

Theme	Category	Subcategory
(1) Iterative Communication & Coordination	1. [Repeatedly sharing the outlook until the end to resolve uncertainty and divergence in the direction of home-based end-of-life care among patients, families, and the palliative care team]	Using the meaning of home death for patients and families as emotional support to overcome care difficulties
		Mutually resolving anxieties among home palliative care team members as the patient’s death approaches
		Establishing early consensus among team members regarding policies and role allocation
		Establishing early consensus between patients/families and healthcare providers regarding how to spend the final days
		Establishing consensus on the home palliative care services to be received/provided between patients/families and healthcare providers
		Sharing strong religious beliefs and confidential family matters with the home palliative care team before intervention
		Preparing a reliable hospitalisation system in case home care becomes unfeasible before intervention
		Responding immediately 24/7 to sudden changes or anxieties that may interrupt home care
		Final confirmation of the intention for home death to solidify the resolve of patients and families
		Raising family awareness of limited time with the patient as their daily activity level declines
		Resolving discrepancies in thoughts about how to spend the final days among patients and families as death approaches
		Continuously monitoring the psychological acceptance of death by patients and families as symptoms worsen
		Sensing the patient’s vitality and proximity to death through changes in appetite, eye strength, and behaviour
		Understanding the patient’s true desires regarding how they wish to live the end of life
	2. [Share real-time information about rapidly changing conditions of cancer patients and their families among all team members to enable immediate feedback in home-based palliative care]	Immediately communicating changes and precautions to the next team member
		Sharing detailed changes in patient/family conditions among the team
		Updating home-based palliative care by consolidating input from team members with limited visits
		Updating team knowledge by sharing patient and family input with infrequent home care members
		Providing patient/family information in a usable format for team members
		Responding to unexpected issues with prompt team meetings
		Adjusting service policies of the multidisciplinary team to match changing needs
		Balancing care provision with changing needs
		Monitoring consistency between needs and care provided
		Preparing for care gaps due to long intervals between visits
		Immediately relieving physical symptoms affecting continued home care through team collaboration
(2) Family Readiness & Relational‑Emotional Support	3. [Replenish the energy required for family caregiving, which is key to achieving home-based end-of-life care]	Monitoring family caregiving capacity regardless of cohabitation status
		Monitoring increasing fatigue and anxiety in family caregivers and their changing attitudes
		Addressing family anxiety about upcoming events in a concrete manner
		Resolving caregiving difficulties and postponed health issues of family members
		Adjusting service times to match family routines and prevent disruption
		Encouraging family confidence in caregiving as death approaches
		Distracting tense family emotions with cheerful conversations
		Reducing the burden of non-routine visits through close communication
	4. [Use conversations tailored to the emotional state of patients and families who are suffering from the proximity of death, to help them feel as calm as possible]	Creating moments for patients to forget the proximity of death through casual conversations
		Easing patient/family tension associated with service visits through friendly attitudes
		Avoiding death-related topics unless requested by the patient
		Accepting amplified suffering and distress empathetically as death approaches
	5. [Reduce psychological distance between cancer patients and their families, who may become mutually isolated due to decreased communication as death approaches]	Promoting communication between patients and families to heal loneliness
		Reducing psychological distance caused by decreased communication
		Creating final moments for patients and families to spend together peacefully
		Encouraging family interaction with patients as their responses diminish
		Supporting realisation of family wishes for the patient considering limited time
		Supporting realisation of patient wishes considering limited time
(3) Proactive Preparedness & Resource Orchestration	6. [Estimate a more effective service plan with minimal costs based on the end-of-life outlook from the early stage of home palliative care]	Gaining patient and family understanding of the overall cost of increasing home palliative care services as death approaches
		Connecting patients and families to systems that minimise expenses for increasing service use
		Planning home palliative care considering the financial situation of patients and families
	7. [Prepare thoroughly to enable immediate and appropriate symptom relief at home for rapidly worsening cancer-related distress]	Preparing medications and administration routes in advance to ensure uninterrupted symptom relief
		Avoiding inappropriate use of opioids due to patient/family anxiety or misunderstanding
		Sharing anticipated symptoms and issues among the team to prepare responses
		Enhancing family coping ability for expected symptoms before death
		Preventing common end-of-life symptoms like constipation and pressure ulcers
	8. [Respect and reflect the patient’s wishes in daily-life support, even as their ability to express those wishes declines due to disease progression]	Providing decision-making opportunities and reflecting patient intentions in life
		Supporting autonomous living by compensating for lost abilities
		Quickly resolving difficulties in eating, toileting, and mobility that affect continued home care
		Prioritising patient wishes over drug efficacy within safe limits except for opioids
	9. [Team members complement and substitute roles beyond their professional boundaries with consensus from patients, families, and the entire team]	Supplementing unavailable care due to manpower shortage with non-specialist team members
		Supplementing care from efficiency/economic perspectives with substitutable team members
		Using informal support to supplement unavailable care
		Teaching families medical procedures to fill care gaps
		Fulfilling responsibilities through teamwork that compensates for each other’s shortcomings
		Fulfilling responsibilities based on required knowledge and skills
		Reorganising the team and care plan continuously to match changing needs
		Forming teams that best meet patient/family needs by understanding strengths of institutions
		Forming responsive teams by understanding other professionals’ availability
		Forming teams that provide support matching estimated time and needs
		Forming collaborative teams by understanding relationships among institutions

Categories are indicated in brackets [ ], and participant narratives are presented in *italics.*

#### (1) iterative communication & coordination (integrating categories 1 and 2)

This theme represents the continuous, cyclical process through which team members, patients, and families repeatedly align their understanding of prognosis, care direction, and emerging needs. It highlights the dynamic coordination and real-time information sharing that enable multidisciplinary teams to sustain home-based end-of-life care despite rapid changes in patient conditions.

Category 1. [Repeatedly sharing the outlook until the end to resolve uncertainty and divergence in the direction of home-based end-of-life care among patients, families, and the palliative care team]

In home-based end-of-life care for patients with terminal cancer, the worsening symptoms and increasing anxiety as death approaches can lead to shifts in perspectives and misunderstandings among patients, families, and team members. These changes may cause uncertainty and divergence from the previously shared care plans, potentially hindering the fulfilment of the patient’s wish to die at home.

To address this, during periods of rapid disease progression or prolonged caregiving in stable phases—when uncertainty is most likely—it is essential to repeatedly confirm the true feelings and wishes of patients and families, share prognostic expectations, and reach consensus on specific care plans. This process helps maintain a shared understanding of the end-of-life trajectory and reduces uncertainty regarding home-based care.

A (Physician): *When the patient returns home with the wish to spend their final days there*,* and their prognosis is short*,* there’s no time to leisurely gather information about the family’s thoughts before the next visit. In such situations*,* we may need to plan everything on the same day—understanding the thoughts of all family members*,* the patient’s wishes*,* and what specific actions need to be taken. It’s important to create a well-prepared care plan and determine priorities based on that plan.*

D (Home Care Nurse): *I say it deliberately. You can probably tell just by looking*,* but I think the time for farewells is approaching. I say things like*,* ‘It seems we’re entering a time when they’re mostly just sleeping.’ Then I ask*,* ‘What did the patient say they wanted at that time?’ or ‘What would you like to do as a family?’ and ‘What should we do?’ Often*,* even without giving direct instructions*,* the family will act in ways they feel are appropriate. It’s a bit tough*,* but I make a point of saying these things.”*

Category 2. [Share real-time information about rapidly changing conditions of cancer patients and their families among all team members to enable immediate feedback in home-based palliative care]

In home-based palliative care, limited visit frequency and divided responsibilities among team members can make it difficult to monitor the rapidly changing conditions of patients and families. This may impede timely and appropriate care strategies.

To address this, team members must continuously exchange up-to-date and detailed information, promptly reviewing and adjusting care plans. This ensures that all members—regardless of their visit schedule—understand the current situation and can respond quickly.

B (Physician): *Interprofessional collaboration should be on the same level. We try to create an environment where communication is easy. For example*,* we use tools like Medical Care Station so that information can be exchanged in real time at any moment.*

K (Certified Care Worker): *We gather information about the patient’s physical condition*,* medications*,* and pain levels. If we notice changes in pain symptoms*,* we report them immediately to the visiting nurse. As patients gradually lose abilities*,* especially those living alone*,* we constantly check what they can still do. When they lose more functions*,* we need to increase services. So*,* we collect information and pass it to the care manager.*

K (Certified Care Worker): *We don’t ask directly about their true feelings—they just slip out naturally. As care workers*,* we’re deeply involved in their daily lives*,* so we pick up on those things. What matters is how well we can catch those feelings and communicate them to the team.*

#### (2) family readiness & relational‑emotional support (integrating categories 3, 4, and 5)

Theme 2 represents the relational and emotional processes through which multidisciplinary teams enhance family caregivers’ psychological readiness, emotional stability, and capacity to remain engaged in home‑based care.

This theme highlights how professionals support families who face escalating emotional burdens, fluctuating acceptance of approaching death, and increasing caregiving strain. It also captures the ways in which providers facilitate meaningful emotional connection, reduce the psychological distance between patients and families, and create conditions that allow both to face the end of life with greater emotional security.

Category 3. [Replenish the energy required for family caregiving, which is key to achieving home-based end-of-life care]

Family caregivers often experience increasing physical and emotional strain owing to the progressive nature of the illness, growing complexity of care, and unexpectedly prolonged caregiving. This can lead to exhaustion and emotional instability, potentially jeopardising home care continuity.

Supporting and encouraging families’ emotional readiness, redistributing caregiving responsibilities according to capacity, supplementing insufficient care, and maintaining daily routines help prevent the depletion of caregiving energy and ensure sustainable home care.

J (Certified Care Worker): *It’s rare for family members to be constantly present. They have jobs and lives*,* right? When we visit*,* it allows them to go to work with peace of mind—not worrying while they’re away. If they know*,* for example*,* ‘The care worker is coming at this time*,*’ they can feel reassured during that period.*

I (Certified Care Worker): *First*,* we start by helping the family understand caregiving. Many of them really don’t know anything. They lack basic knowledge—like which type of pad to use. We start from there*,* and then move on to things like meals.*

Category 4. [Use conversations tailored to the emotional state of patients and families who are suffering from the proximity of death, to help them feel as calm as possible]

Patients with terminal illness as well as their families often become increasingly aware of death through interactions with palliative care team members and discussions about dying. This heightened awareness can intensify existential distress and make it difficult for them to spend their final days peacefully at home.

To address this, care providers should respond with full empathy and presence when patients or families openly express their emotional pain related to death. Conversely, when such distress remains internalised, providers should avoid death-related topics and instead engage in light-hearted conversation or show familiarity to create moments of emotional relief. These approaches help maintain emotional stability and comfort for both patients and families.

F (Home Care Nurse): *Timing is really important. Sometimes the patient seems calm. If we talk about death too early*,* like saying ‘death is inevitable*,*’ they might feel like*,* ‘I’m not in that situation yet.’ So*,* I’m very careful when it comes to talking about death. When I do mention it*,* I watch their facial expressions closely—they often tense up. I try to gauge the right moment. I believe it’s okay to bring up the topic when the time truly comes*,* but I avoid using words that make them feel death is near too early on.*

Category 5. [Reduce psychological distance between cancer patients and their families, who may become mutually isolated due to decreased communication as death approaches]

As death nears, patients may become less responsive, and families may struggle with how to interact with their loved ones. This can reduce meaningful exchanges, even while living together, resulting in emotional distance and missed opportunities to share final moments.

Care providers promote emotional and physical closeness, create private time for them to feel safe and connected, and support the realisation of mutual wishes. These efforts help foster empathy and understanding, allowing patients and families to spend their remaining time together in a fulfilling way.

D (Home Care Nurse): *I wondered if they were still having conversations*,* if they were still laughing together. But as I got involved*,* I felt like emotions weren’t being expressed. Like*,* ‘Did you eat everything?’ and just a vague ‘Hmm…’ in response. So*,* I thought maybe they needed a moment to let their emotions out. I tried to figure out why they couldn’t express themselves. I realised—it’s because they didn’t want to accept death. I thought*,* maybe if they could cry*,* they could start to accept it. And maybe speaking to each other would help them find closure. All I saw was someone trying to escape the reality of the situation. In this case*,* I felt that crying might be the key to bringing their hearts closer together.*

#### (3) proactive preparedness & resource orchestration (integrating categories 6, 7, 8, and 9)

Theme 3 captures the proactive and anticipatory practices through which multidisciplinary teams prepare medical, practical, and emotional resources in advance to ensure timely and appropriate responses to rapidly changing conditions in home-based end-of-life care.

This theme emphasises planning, early symptom anticipation, coordination of available services, and flexible role-sharing to prevent crises and sustain home care. It also reflects the deliberate orchestration of financial, logistical, and caregiving resources that enables respect for patients’ wishes to remain at home, even under resource-limited conditions.

Category 6. [Estimate a more effective service plan with minimal costs based on the end-of-life outlook from the early stage of home palliative care]

In home-based end-of-life care, the cumulative costs of cancer treatments and palliative care services can generate a financial burden. As the disease progresses and the need for end-of-life services increases, rising expenses and financial anxiety may result in hesitation to use necessary services, potentially delaying essential care.

To mitigate this, it is important to anticipate care needs from the outset of home palliative care and devise strategies to minimise overall costs. Connecting patients and families to available financial support systems allows them to receive necessary care without undue worry. This approach enables effective and efficient home palliative care that is mutually acceptable to both the patient/family and the care team.

M (MSW): *My involvement is far from a routine administrative explanation. I listen carefully to what the patient and family are struggling with—whether it’s the severity of the illness or anxiety about what lies ahead. I explain the financial burden and try to provide concrete figures about the maximum out-of-pocket costs. I work to eliminate vague fears like ‘How much will the bill be after the patient passes away?’ Once those worries are eased*,* they can feel more comfortable calling the doctor or using medications without hesitation.*

Category 7. [Prepare thoroughly to enable immediate and appropriate symptom relief at home for rapidly worsening cancer-related distress]

In home-based end-of-life care, the absence of medical professionals, limited supplies, and the inadequate caregiving capacity of family members can delay or hinder responses to rapidly worsening symptoms, potentially exacerbating the patient’s suffering. Anticipating symptom progression, securing necessary supplies, and educating family members ensure that immediate and appropriate care can be provided even during sudden deterioration.

B (Physician): *We make preparations to allow the patient to stay at home. We do this in advance*,* step by step. For example*,* if symptom relief is needed*,* we explain about possible symptoms like ileus and*,* depending on the case*,* we prepare the necessary medications in advance. We aim to prevent emergencies from becoming emergencies. It’s about preparing both the physical resources and the emotional readiness.*

Category 8. [Respect and reflect the patient’s wishes in daily-life support, even as their ability to express those wishes declines due to disease progression]

As cancer progresses, patients may experience rapid declines in the ability to perform daily activities and express their wishes, which can threaten autonomy and dignity. To address this, care providers should continuously confirm the patient’s intentions regarding daily self-care, supplement activities the patient can no longer perform, and prioritise the realisation of their wishes—even if doing so involves some risk to their health. These efforts ensure that patients can continue living at home in accordance with their values and preferences until the end.

D (Home Care Nurse): *What matters is how well I can capture the patient’s wishes and how prepared I am to fulfil them. I think it’s important to have a ‘trump card’ ready—something that allows me to make their wishes come true. When I’m able to do that*,* I feel it was a good outcome.*

Category 9. [Team members complement and substitute roles beyond their professional boundaries with consensus from patients, families, and the entire team]

In resource-limited regions or during rapid disease progression, home-based palliative care teams may face shortages of necessary professionals or encounter situations where specialised roles alone cannot meet diverse patient and family needs. Therefore, flexible team composition and role-sharing across disciplines are essential.

By forming consensus among patients, families, and team members, professionals can step beyond their formal roles to fill gaps in care. This collaborative approach ensures timely and appropriate support, even in resource-constrained settings.

K (Certified Care Worker): *Even if I only visit every two weeks*,* I try to accurately understand the patient’s condition and pain levels. If I can observe and report that to the nurse*,* maybe the nurse doesn’t need to visit as often. In that sense*,* I think care workers can sometimes take on that role.*

## Discussion

This qualitative descriptive study elucidated how integrated transdisciplinary teams sustain home-based end-of-life care for patients with terminal cancer in resource-limited settings. Through reflexive thematic analysis, three interrelated themes were identified: (1) Iterative Communication & Coordination, (2) Family Readiness & Relational–Emotional Support, and (3) Proactive Preparedness & Resource Orchestration. Together, these themes constitute a coherent practice architecture through which patients’ wishes for home death are realised (Table 2).

The identified themes do not represent discrete or isolated practices; rather, they illustrate a dynamic and mutually reinforcing process by which teams continuously align prognostic understanding, strengthen caregiver readiness, and proactively organise medical, practical, and financial resources. Although previous studies have demonstrated the effectiveness of multidisciplinary team approaches to home palliative care [14, 15], and qualitative research has illuminated the collaborative practices underpinning high-quality home-based palliative care [29], this study extends existing knowledge by systematically articulating how transdisciplinary collaboration—including the contributions of welfare professionals—functions beyond formal professional boundaries in everyday practice.

### Iterative communication & coordination

The first theme highlights the central role of repeated alignment of prognostic perspectives and real-time information sharing among patients, families, and team members. Previous studies have demonstrated that shared decision-making and effective communication are key determinants of quality in home palliative care [14, 15, 22, 23]. Our findings build on this literature by showing that such alignment is not a one-off event, but an iterative process that intensifies as death approaches and as patients’ conditions and family perspectives fluctuate.

A notable contribution of this study is the demonstration of how information derived from daily-life support—often gathered by care workers or CMs—is translated into clinical decision-making. These professionals, who are closely involved in patients’ daily lives, play a crucial role in detecting subtle physical or emotional changes and communicating them to the wider team. This finding is consistent with prior research emphasising the importance of horizontal professional relationships and information and communications technology (ICT)-supported information sharing in palliative care teams [30, 31].

### Family readiness & relational–emotional support

The second theme foregrounds family caregiver readiness as a core mechanism underpinning the feasibility of home death. While caregiver burden and psychological distress have been widely documented as barriers to sustained home care [10, 32], this study advances understanding by detailing how multidisciplinary teams actively and continuously assess and strengthen the psychological and practical capacities of caregivers.

Teams supported families by replenishing their caregiving energy through service adjustment, respite-like support, and emotional reassurance, while carefully tailoring conversations according to caregivers’ fluctuating emotional states. Practices such as modulating the timing and language of discussions about death and facilitating emotional expression between patients and families align with evidence on dignity therapy and end-of-life psychological support [33–35]. Collectively, these findings suggest that caregiver readiness should not be conceptualised as a fixed prerequisite for home death, but as a dynamic state co-constructed through ongoing team engagement.

### Proactive preparedness & resource orchestration

The third theme captures anticipatory practices through which teams prepare for symptom escalation, financial strain, and potential care gaps before the occurrence of crises. The importance of anticipating symptom progression and preparing responses in advance has been emphasised in prior palliative care research [6, 7, 36], and our findings further demonstrate how such preparedness contributes to the continuity of home care.

This is consistent with evidence indicating that financial burden can adversely affect the quality of life of patients and families [10], and that addressing economic concerns is an important component of comprehensive palliative care planning [36]. Moreover, in contexts characterised by workforce shortages or uneven resource distribution, flexible role substitution across professional boundaries emerges as a pragmatic and effective strategy. This form of role-sharing, grounded in consensus among patients, families, and team members, reflects transdisciplinary collaboration rather than a dilution of professional expertise [19, 21, 31].

### Cultural and institutional context of Japan

The practices identified in this study can be better understood when situated within the context of Japanese culture. In Japan, family-centred decision-making is common, and patients frequently rely on family members to express preferences related to end-of-life care [37]. This cultural norm amplifies the need to repeatedly share prognostic perspectives and align expectations among patients, families, and healthcare providers. Furthermore, Japan’s high-context communicative style means that emotional distress and care preferences are often conveyed implicitly rather than verbally. Within this cultural context, practices such as repeated sharing of prognostic outlooks and indirect emotional attunement become particularly critical, as patients and families may avoid explicit discussions of death while still requiring a shared understanding to sustain home care. Consequently, professionals who engage with patients in their daily lives, such as visiting nurses and care workers, play a particularly important role in detecting subtle cues and communicating them to the wider team [3, 35]. Finally, societal expectations that families should provide substantial caregiving, combined with the structure of the long-term care insurance system, shape both the emotional burden and the practical challenges of caregiving. In addition, the structural separation between medical insurance and long-term care insurance necessitates intentional coordination across medical and welfare sectors, reinforcing the importance of transdisciplinary collaboration [15, 16, 38].

These cultural and institutional characteristics help explain why practices such as caregiver support, financial planning, and flexible role-sharing emerged as essential components of high-quality, home-based palliative care in the present study.

### Implications for practice, education, and policy

The three themes identified in this study can serve as shared practice indicators for multidisciplinary teams providing home-based palliative care. Practices such as real-time information sharing and anticipatory symptom management may help reduce unnecessary emergency admissions and support continuity of care at home [14, 36].

To operationalise the empirically derived themes identified in this study, we propose three practice-oriented modules that translate transdisciplinary collaboration into actionable routines within home-based palliative care. These modules are illustrative, practice-informed examples derived from the identified themes and should not be viewed as prescriptive interventions.

First, a module on Iterative Communication & Coordination institutionalises the repeated alignment of prognostic understanding and care goals through ICT-supported team huddles, structured real-time reporting, and simulations of rapid role coordination during sudden clinical or psychosocial changes. Grounded in practices described in Categories 1 and 2, this module addresses the need for continuous integration of information from professionals with differing visit frequencies, thereby reducing uncertainty and preventing fragmentation of care.

Second, a module focusing on Family Readiness & Relational–Emotional Support systematically strengthens the psychological and practical capacities of caregivers across the illness trajectory. Drawing on Categories 3 to 5, this module incorporates the assessment of caregiver readiness, role-play exercises on the timing and language of end-of-life conversations, and case-based reflection on facilitating emotional expression without imposing premature death awareness. By reframing caregiver readiness as a dynamic and co-constructed outcome rather than a fixed prerequisite, this module supports sustained home care and mitigates caregiver exhaustion that often precipitates institutionalisation.

Third, a module on Proactive Preparedness & Resource Orchestration incorporates anticipatory planning and flexible role-sharing into routine practice. Based on Categories 6 to 9, this module includes rapid-response deterioration scenarios, early financial planning reviews, and role-translation workshops in which professionals clarify core and adjacent tasks and negotiate safe substitution boundaries. By positioning preparedness as a collective team responsibility, this module enables timely symptom relief, respects patients’ wishes in daily-life support, and compensates for workforce shortages in resource-limited settings.

Collectively, these modules provide a structured pathway for embedding transdisciplinary practices into daily care. They offer concrete implications for clinical practice, inform the design of targeted interdisciplinary education, and highlight policy-relevant strategies, such as supporting role flexibility and strengthening information infrastructure, that may enhance equity and sustainability in community-based palliative care.

### Methodological limitations and future research directions

This study has some methodological limitations. First, data were collected from a single region in Japan, and the sample comprised 13 multidisciplinary professionals with extensive experience in home-based palliative care. Because this study employed a qualitative descriptive design informed by reflexive thematic analysis, the findings are not intended to be statistically generalisable or reproducible in a positivist sense. Instead, their value lies in their analytical transferability to similar contexts.

Second, the primary interviewer’s extensive clinical experience facilitated rapport and rich data collection but also shaped the interpretive lens during analysis. Although reflexive practices and team-based discussions were used to enhance analytical transparency, the themes represent one possible interpretive construction rather than a fully reproducible account. In addition, the use of a semi-structured interview guide and reliance on self-reported data may have influenced participants’ narratives.

Finally, although interviews were transcribed verbatim and professionally translated, some linguistic and cultural nuances may not be fully reflected in the English excerpts. As this study aimed to structure practical knowledge rather than develop formal theory, the findings should be interpreted as exploratory and context-dependent. Future research should examine the applicability of these practices across different cultural, organisational, and healthcare contexts.

## Conclusion

This qualitative descriptive study provides a practice-oriented understanding of how transdisciplinary teams enable home-based end-of-life care for patients with terminal cancer. The study sought to elucidate concrete and transferable practices through which integrated teams respond to patients’ wishes, support family caregivers, and coordinate care in the community.

The three interrelated themes—(1) Iterative Communication & Coordination, (2) Family Readiness & Relational–Emotional Support, and (3) Proactive Preparedness & Resource Orchestration—collectively describe a coherent practice architecture that sustains home care until death. The nine identified categories illustrate how teams dynamically integrate medical, welfare, and daily‑life support practices while flexibly sharing roles to compensate for workforce limitations and uneven resource distribution.

Although grounded in a specific regional and cultural context in Japan, the findings offer analytically transferable insights for other settings facing similar challenges in home-based palliative care. By making tacit transdisciplinary practices explicit, this study contributes a practice-informed framework that can inform interdisciplinary education, guide service development, and support policy efforts aimed at strengthening equitable and sustainable community-based palliative care.

## Supplementary Information

Additional file 1: COREQ Compliance Table. The 32-item reporting guidelines for qualitative research and the correspondence table with this study are presented.

Additional file 2: Interview guide. Semi-structured interview guide used to explore high-quality home-based palliative care practices by multidisciplinary teams. The guide includes prompts for participants to recall memorable cases where patients with terminal cancer were able to die at home in accordance with their wishes and where patients, families, and team members were satisfied with the care provided. Topics covered include the care process from the time the patient expressed their wish to receive care at home, through the home death, to bereavement care, as well as team collaboration, management of challenges such as manpower shortages, and essential practices for achieving a satisfactory home death.

## Data Availability

The datasets generated and/or analysed during the current study will be available from the corresponding author upon reasonable request. Owing to the potentially identifiable nature of detailed qualitative data, a complete dataset is not available. Access to patient-identifiable data will be restricted to members of the research team. Data and results will not be used for secondary analyses in future research and/or for studies involving modified or different research questions.

## Abbreviations

CM(s): Care Manager(s)
COREQ: Consolidated Criteria for Reporting Qualitative Studies
ICT: Information and Communications Technology
MSW(s): Medical Social Worker(s)
