# Supplementary material for: Effective practices and transdisciplinary team-based approaches in home palliative care for terminal cancer patients: a qualitative descriptive study
Source: BMC Palliat Care. 2026 Apr 17;25:177. doi: 10.1186/s12904-026-02102-3 (PMC13277223; doi:10.1186/s12904-026-02102-3)
Supplement: Supplementary file 1 — Additional file 1: COREQ Compliance Table. The 32-item reporting guidelines for qualitative research and the correspondence table with this study are presented. [file 12904_2026_2102_MOESM1_ESM.docx]

**Additional file 1. COREQ Compliance Table**

This appendix presents the compliance of the study with the Consolidated Criteria for Reporting Qualitative Research (COREQ) 32-item checklist. Page references correspond to sections in the manuscript.

*Table A1. Compliance of the study with the COREQ 32-item checklist*

| **No.** | **COREQ Item** | **Compliance/Description** | **Page Reference** |
| --- | --- | --- | --- |
| 1 | Interviewer credentials | Roles and qualifications described | Methods – Analysis　P13 |
| 2 | Occupation | Authors’ affiliations listed | Title page　P1 |
| 3 | Gender of data collectors | Reported: The interviewer was a female registered nurse. | Results – Characteristics　P16 |
| 4 | Experience and training | Analytical experience indicated | Data collection　P12 |
| 5 | Relationship established | No prior relationship existed | Data collection　P12 |
| 6 | Participant knowledge of interviewer | Study independence explained | Data collection　P11 |
| 7 | Interviewer characteristics | Passive yet attentive stance noted | Ethical considerations　P15 |
| 8 | Theoretical framework | Qualitative descriptive design; thematic analysis | Methods – Study design　P8 |
| 9 | Participant selection | Criteria based on experience and cases | Methods – Participant selection　P8 |
| 10 | Sampling | Purposive sampling | Methods – Participant selection　P8 |
| 11 | Method of approach | Invitation and consent procedures described | Participant selection/Ethics　P8, P15 |
| 12 | Sample size | 13 professionals | Results – Characteristics　P16 |
| 13 | Non-participation | No eligible participants declined | Participant selection　P10 |
| 14 | Setting of data collection | Private environment; web option | Data collection　P11 |
| 15 | Presence of non-participants | No non-participants were present; interviews were conducted in private settings | Result – Characteristics of the participants P16  Methods – Ethical considerations　P15 |
| 16 | Description of sample | Profession, sex, age, and years of experience described | Results – Table 1　P47 |
| 17 | Interview guide | Semi-structured guide provided | Additional file 2 |
| 18 | Repeat interviews | **Reported:** No repeat interviews were conducted. All necessary data were obtained during a single interview session with each participant. | Result – Characteristics of the participants P16 |
| 19 | Audio/visual recording | Audio recorded and transcribed verbatim | Methods – Ethical considerations　P15 |
| 20 | Field notes | Field notes not used | Methods – Data collection　P12 |
| 21 | Duration | Mean 72.8 minutes | Results – Characteristics　P17 |
| 22 | Data saturation | In line with the principles of reflexive thematic analysis, sample adequacy was assessed based on the richness and depth of the data rather than the achievement of data saturation. | Results – Characteristics　P16 |
| 23 | Transcripts returned | **Reported:** Transcripts were not returned to participants. This decision was made to reduce participant burden and to maintain focus on the aim for descriptive analysis rather than member‑verified interpretation. | Methods – Analysis　P13 |
| 24 | Number of data coders | Independent coding by two researchers | Methods – Analysis　P13 |
