# Supplementary material for: Effective practices and transdisciplinary team-based approaches in home palliative care for terminal cancer patients: a qualitative descriptive study
Source: BMC Palliat Care. 2026 Apr 17;25:177. doi: 10.1186/s12904-026-02102-3 (PMC13277223; doi:10.1186/s12904-026-02102-3)
Supplement: Supplementary file 2 — Additional file 2: Interview guide. Semi-structured interview guide used to explore high-quality home-based palliative care practices by multidisciplinary teams. The guide includes prompts for participants to recall memorable cases where patients with terminal cancer were able to die at home in accordance with their wishes and where patients, families, and team members were satisfied with the care provided. Topics covered include the care process from the time the patient expressed their wish to receive care at home, through the home death, to bereavement care, as well as team collaboration, management of challenges such as manpower shortages, and essential practices for achieving a satisfactory home death. [file 12904_2026_2102_MOESM2_ESM.docx]

**Additional file 2. Interview Guide**

1. Basic Information

・Please tell me your age.

・Please describe your professional qualifications.

・How many years of experience do you have in your primary professional qualification?

・Please describe the characteristics of the institution you belong to (e.g., medical corporation, independent clinic, social welfare corporation).

・In the past year, how many patients with terminal cancer have you cared for at home until death?

2. Interview Questions

Please share a memorable case of high-quality end-of-life care at home from your past experiences. “High-quality end-of-life care” refers to a home death that was perceived as satisfactory by the patient, their family, and the involved multidisciplinary team.

a) What type of team members were involved in the care of this patient?

b) What practices did you implement to realise home-based end-of-life care for this patient? Please describe the process in order, from the time the patient expressed their wish to receive care at home, through the home death, to bereavement care. What were the reactions of the patient and their family at each stage?

c) How did you collaborate with the team members during this case? What were the team members’ attitudes, thoughts, roles, and methods of collaboration?

d) Looking back on this case, what practices do you think were successful, and why? Were there any practices that did not go well? If so, why do you think they were not successful, and what could have been done differently?

e) Based on your experience, what do you consider important when providing care and collaborating with a team to support terminal cancer patients at home? Why is that important?

f) Based on your experience, what types of practices and care do you think are necessary as a multidisciplinary team?

g) What are your thoughts on the ideal structure and collaboration of a multidisciplinary team to support home-based end-of-life care for terminal cancer patients?

h) In situations where necessary services cannot be provided because of financial constraints or lack of manpower (e.g., absence of professionals to fulfil essential roles), how does your team respond to meet the needs of patients and their families?
